# Do Trained Practice Nurses Apply Motivational Interviewing Techniques in Primary Care Consultations?

**DOI:** 10.4021/jocmr1120w

**Published:** 2012-11-11

**Authors:** Janneke Noordman, Inge van Lee, Mark Nielen, Hans Vlek, Trudy van Weijden, Sandra van Dulmen

**Affiliations:** a NIVEL, Netherlands Institute for Health Services Research, PO Box 1568, 3500 BN Utrecht, The Netherlands; b Primary Care Centre Tiel (Eerstelijns Centrum Tiel), Dodewaardlaan 9, 4006 EA Tiel, The Netherlands; c Maastricht University, School for Public Health and Primary Care (CAPHRI), Department of General Practice, P.O. Box 616, 6200 MD Maastricht, The Netherlands; d Radboud University Nijmegen Medical Center, Department of Primary and Community Care, Geert Grooteplein Noord 21, 6500 HB Nijmegen, The Netherlands; e Department of Health Science, Buskerud University College, Drammen, Norway

**Keywords:** Communication, Life style, Nurses, Prevention, Primary health care

## Abstract

**Background:**

Reducing the prevalence of unhealthy lifestyle behaviour could positively influence health. Motivational interviewing (MI) is used to promote change in unhealthy lifestyle behaviour as part of primary or secondary prevention. Whether MI is actually applied as taught is unknown. Practice nurses’ application of motivational interviewing in real-life primary care consultations was examined. Furthermore, we explored if (and to what extent) practice nurses adjust their motivational interviewing skills to primary versus secondary prevention.

**Methods:**

Thirteen Dutch practice nurses, from four general practices, trained in motivational interviewing participated, 117 adult patients visiting the practice nurse participated, 117 practice nurse-patient consultations between June and December 2010 were videotaped. Motivational interview skills were rated by two observers using the Behaviour Change Counselling Index (BECCI). Data were analyzed using multilevel regression.

**Results:**

Practice nurses use motivational interviewing techniques to some extent. Substantial variation was found between motivational interviewing items. No significant differences in the use of motivational interviewing between primary and secondary prevention was found.

**Conclusions:**

Motivational interviewing skills are not easily applicable in routine practice. Health care providers who want to acquire motivational interview skills should follow booster sessions after the first training. The training could be strengthened by video-feedback and feedback based on participating observation. A possible explanation for the lack of differences between the two types of prevention consultations may be the gain to help patients in primary consultations by preventing complications equals the necessity to help the disease from aggravating in secondary prevention.

## Introduction

The World Health Organization advocates the integration of strategies to prevent and manage lifestyle-related chronic conditions in primary health care [1]. Primary health care is a suitable context to identify and address behavioral risk factors [2-4] since utilisation by the general population is high [5, 6]. Furthermore, interventions aimed at behavioral change often require regular health care contacts [4]. Even though the community added value of primary care preventive activities is generally accepted and broadly advocated, the quality of the actual delivery process during everyday healthcare visits is usually taken for granted, and implementation checks are scarce.

The majority of preventive activities in primary care are delivered by a physician or general practitioner (GP). Generally, GPs make the initial diagnosis, initiate the treatment and facilitate overall continuity of care [7]. Yet, discussing lifestyle and referring to programmes promoting lifestyle can be well performed by a practice nurse (PN) [8-10]. Previous research shows that PNs monitor patients with chronic conditions such as type 2 diabetes [11], provide patients with lifestyle advice and guide them during smoking cessation and weight reduction [12]. The level of professional autonomy of nurses varies both within and across countries, but is generally high [13].

Due to GP’s lack of time, PNs might be even more suitable for promoting healthy lifestyles than GPs [14, 15]. As PNs spend more time on counseling patients than GPs [16, 17], they may be more oriented towards counseling and other behavioral techniques of importance for the prevention of lifestyle-related chronic conditions.

In the USA, the UK, and beyond, promising results have been reported in changing an unhealthy lifestyle using motivational interviewing (MI) techniques [18-20]. MI is a patient-centered directive approach to enhance intrinsic motivation to behavioral change by helping patients explore and resolve ambivalence between desired behavior and actual behavior [21, 22]. It focuses on what patients can do to improve their health, as opposed to health care providers telling them what to do. MI has shown to produce positive health behavior change and maintenance [23]. However, there are other behavior change approaches that show similar positive health outcomes [24, 25].

Previous research into MI has focused predominantly on determining whether or not MI is more effective for changing behavior than other interventions [6, 26, 27]. Little is, however, known about the actual use of MI techniques in everyday consultations in general or by PNs specifically [28-30].

The aim of the present study is to assess whether PNs apply MI techniques in consultations and if (and to what extent) they adjust their MI skills to primary or secondary prevention of lifestyle-related diseases. A primary prevention consultation aims to prevent the development of a chronic disease such as heart and vascular disease, type 2 Diabetes Mellitus and Chronic Obstructive Pulmonary Disease (COPD), and therefore focuses mainly on avoiding health risks. Secondary prevention consultations focus on people already affected by a chronic disease and attempt to enhance the patient’s autonomy, minimize the consequences of the disease and prevent the disease from aggravating. It may be important to distinguish between these two types of prevention consultations as they may require different communication strategies; in primary prevention, PNs may need more time and effort to motivate the patient compared to secondary prevention which may also be reflected in longer visits.

## Materials and Methods

The study has an observational design; real-life consultations between PNs and patients within primary care were observed.

### Participants

Thirteen PNs from four practices trained in MI participated. All PNs from three practices were approached by contacting the GPs of these practices, who (except for one practice) participated in an earlier study [31]. GPs from one other practice (health care center) contacted us for participation of all of their PNs. PN’s prior MI training varied between 1/2 day to six half days. On average PNs had four years of working experience (SD: 2.47, range: 9 months - 9 years and 11 months).

Adult patients scheduled for an appointment with the PN between June and December 2010 were eligible for inclusion. We included approximately ten patients per PN.

### Procedure

Consecutive consultations were videoed using an unmanned digital camera located unobtrusively in the PN’s consulting room for one or two random days. The aim was to record ten routine consultations per PN. All the participating PNs provided care to a mixed group of patients e.g. with type 2 diabetes, COPD (secondary prevention), or high risk patients (primary prevention) with e.g. hypertension, counseling on smoking. Discussing patient’s lifestyle behavior was a potential component of the consultations, which a PN or patient could bring up.

Patients were approached by a researcher in the waiting room. They were asked to give written informed consent and to complete a short questionnaire about sociodemographics and lifestyle behaviors (smoking, alcohol use and physical activity).

PNs also gave written informed consent before the video-recording of the consultations and completed a short questionnaire after each consultation about the perceived complaints and diseases of patients. PNs were aware that the study focused on their application of MI skills. Patients were told that analyses would focus on communication skills of PNs in general. Participants could withdraw their consent at any time; no one did.

This study was performed according to Dutch privacy legislation. The privacy regulation was approved by the Dutch Data Protection Authority. According to Dutch legislation, approval by a medical ethics committee was not required for this observational study.

### Observations and reliability

The videotaped consultations were rated by two observers independently. For each consultation the application of MI was coded, using the Behaviour Change Counselling Index (BECCI) checklist [32, 33]. The BECCI contains eleven, five-point Likert-scaled items related to the practitioners’ behaviors and MI techniques, ranging from ‘not at all’ to ‘a great extent’ (see Appendix 1). These items are subdivided into four domains: agenda setting and permission seeking (two items, Cronbach’s α = 0.61); the why and how of change in behavior (five items, α = 0.70); the whole consultation (three items, α = 0.69); and talk about targets (one item).

Additionally, as part of BECCI, PNs’ speaking time is assessed with a separate item. In line with MI, a practitioner is expected to talk no more than 50% of the consultation time. Therefore, speaking time is divided into ‘consultation with PNs’ speaking time half of the time or less’ versus ‘more than half of the time’. In previous research, BECCI has demonstrated acceptable levels of reliability, validity and sensitivity to detect change [33-35]. To assess interrater reliability, 21 consultations were rated by both observers, resulting in a sufficiently high average Kappa score [36] of 0.81 (range 0.72 - 1.00).

### Statistical analysis

First, characteristics of the patients in the two prevention groups were calculated. Differences between the groups were tested using independent t-test for continuous variables and chi-square test for dichotomous and categorical variables. Second, the average scores for the separate BECCI-domains, the BECCI total, speaking time and consultation length are compared between the two groups with an independent t-test and a chi-square test. Third, multilevel linear regression analyses with a random intercept were performed to determine the association between the four domain scores of the BECCI, BECCI total score and consultation length (dependent variables) with the types of prevention consultations (model 1). This means that we created a separated model for every dependent variable with the type of prevention consultation. The multilevel technique was used to correct for clustering of patients within PNs [37]. Thereafter, model 1 (for every dependent variable) was corrected for confounding in two steps. Firstly, patients’ social demographic characteristics - age, gender, marital status, educational background and ethnicity - were added to the model (model 2). Secondly, patients risk factors - smoking, alcohol use and physical activity - were added to model 2 (model 3). Interaction terms were added to model 3 to test for potential effect modification.

Finally, the association between the speaking time of the PN and the types of prevention consultations was analyzed with multilevel logistic regression analyses with a random intercept using the second order PQL method (model 1). Due to the low number of patients in the primary prevention group (n = 39), this association could only be corrected for age and gender (model 2). The analyses were performed in Stata 11 (www.stata.com) and MLwiN [38].

## Results

### Subjects

A total of 117 consultations were analyzed; 78 were secondary prevention consultations, of which 58 with patients with type 2 diabetes, and 39 were aimed at primary prevention, e.g. hypertension (n = 18), high cholesterol (n = 2), impaired glucose tolerance (n = 4) or combinations of these. Patient’s non-response rate was 6%. We excluded four patients receiving Cardiovascular Risk Management (CVRM) because CVRM-consultation can be classified as a primary as well as a secondary prevention consultation at the same time. The characteristics of the two groups are depicted in Table 1.

**Table 1:** Baseline Characteristics of the Patients in Both Groups

	Primary prevention n = 39	Secondary prevention n = 78	P-value
Mean age in years (SD; range)	64.2 (12.1; range: 38.0 - 84.6)	64.5 (11.9; range: 29.4 - 86.2)	0.90
Men (%)	43.6	47.4	0.69
Educational level (%)			
low	22.2	36.5	0.16
middle	69.4	50.0	
high	8.3	13.5	
Married/living together (%)	83.3	71.4	0.17
Dutch ethnicity (%)	89.7	84.2	0.42
Smoking; daily/now and then (%)	26.3	28.4	0.82
Alcohol use; daily/now and then (%)	76.3^*^	52.7^*^	0.02
Meets recommended physical exercise (%)	61.1	56.2	0.62

^*^ Significant difference between primary prevention and secondary prevention, χ^2^ test, (P < 0.05).

### The application of MI techniques

Table 2 shows the average scores on the separate BECCI-domains, BECCI mean sum score, speaking time and consultation length. PNs scored highest in Domain 1, with an average score of 2.2. The underlying item about inviting the patient to talk about behavior change had a mean score of 1.5, the item ‘demonstrating sensitivity to talking about other issues’ had a mean score of 2.8. The participating PNs tend to give patients a choice in what to talk about, but they scored low on asking patients about their willingness to talk about their behavior.

**Table 2:** Mean and Standard Deviation BECCI-Domains, BECCI Mean Sum Score, Consultation Length and Speaking Time (%) in Both Groups

	Primary and secondary prevention	Primary prevention	Secondary prevention	P-value^*^
	n = 117, Mean (SD)	n = 39, Mean (SD)	n = 78, Mean (SD)	
Domain 1 Agenda setting and permission seeking	2.2 (0.63)	2.2 (0.55)	2.2 (0.67)	0.80
Item 1 Practitioner invites the patient to talk about behavior change.	1.5 (0.91)	1.5 (0.82)	1.6 (0.95)	0.83
Item 2 Practitioner demonstrates sensitivity to talking about other issues.	2.8 (0.57)	2.8 (0.52)	2.8 (0.60)	0.82
Domain 2 The why and how of change in behavior	1.7 (0.65)	1.7 (0.54)	1.6 (0.70)	0.86
Item 3 Practitioner encourages the patient to talk about current behavior or status quo.	2.8 (0.62)	2.8 (0.51)	2.7 (0.67)	0.53
Item 4 Practitioner encourages the patient to talk about behavior change.	1.8 (1.10)	2.0 (1.09)	1.7 (1.10)	0.15
Item 5 Practitioner asks questions to elicit how the patient thinks and feels about the topic.	1.6 (1.10)	1.6 (1.09)	1.6 (1.11)	0.81
Item 6 Practitioner uses empathic statements when the patient talks about the topic.	0.8 (1.07)	0.7 (0.93)	0.9 (1.13)	0.50
Item 7 Practitioner uses summaries to bring together what the patient says about the topic.	1.3 (1.00)	1.2 (0.79)	1.4 (1.09)	0.36
Domain 3 The whole consultation	2.0 (0.73)	2.1 (0.69)	2.0 (0.75)	0.34
Item 8 Practitioner acknowledges challenges about behavior change that the patient faces.	0.9 (1.11)	0.9 (1.07)	0.8 (1.14)	0.62
Item 9 When practitioner provides information, it is sensitive to the patient concerns and understanding.	3.1 (0.81)	3.2 (0.70)	3.1 (0.87)	0.64
Item 10 Practitioner actively conveys respect for the patient choice about behavior change.	2.1 (0.86)	2.3 (0.86)	2.0 (0.85)	0.19
Domain 4: Talk about targets	1.9 (0.81)	2.0 (0.82)	1.9 (0.80)	0.38
Item 11 Practitioner and the patient exchange ideas about how the patient could change current behavior.	1.9 (0.81)	2.0 (0.82)	1.9 (0.80)	0.38
BECCI mean sum score	1.9 (0.60)	2.0 (0.52)	1.9 (0.63)	0.57
Consultation length (minutes)	22.1 (10.02)	21.0 (9.25)	22.5 (10.40)	0.41
Consultations with PN speaking time half of the time or less (%)	82.0	84.6	80.8	0.61

Scale of the domains: 0 = Not at all, 1 = Minimally, 2 = To some extent, 3 = A good deal, 4 = A great extent. ^*^No significant difference between primary prevention and secondary prevention, T test, (P < 0.05).

Domain 2 showed the lowest average score of 1.7. The underlying item about encouraging the patient to talk about current behavior or status quo had an average score of 2.8. This suggests that PNs regularly ask open questions and/or use empathic listening statements, but fail to meet the other four items belonging to domain 2 (notably: the lack of using empathic statements when the patient talks about the topic averaged 0.8).

Domain 3 had a mean score of 2.0. The average score of 3.1 for the item about providing information which is sensitive to patient’s concerns and understanding is high compared to the item ‘acknowledges challenges about behavior change that the patient faces’ (mean 0.9). PNs try to understand what the patient knows and wants to know, but focus insufficiently on the personal strengths of the patient while facing behavioral changes.

Domain 4 consists of one item ‘exchanging ideas about how the patient could change current behavior’ and has an average score of 1.9.

In line with BECCI, practitioners should talk no more than 50% of the time. In 18% of the consultations PNs talked more than 50% of the time.

The duration of consultations varied between 4.6 minutes and 46.7 minutes, with a mean duration of 22.5 minutes (sd = 10.02). There is a significant relationship between the consultation length and PNs use of MI techniques. However, the effects (r = 0.02) are very small (results of regression coefficients not shown).

### Differences between primary and secondary prevention consultations

Table 3 presents the results of the multilevel analyses with MI techniques and consultation length as dependent variables and the two types of prevention consultations as independent variables.

**Table 3:** Regression Coefficients of the Type of Prevention Consultation on the Various Domains, the BECCI Mean Sum Score and Consultation Length

	Model 1: regression coefficient (95%CI)	Model 2 (demographic characteristics): regression coefficient (95%CI)	Model 3 (demographic characteristics + risk factors): regression coefficient (95%CI)
Domain 1 Agenda setting and permission seeking	0.11 (-0.26 - 0.29)	0.08 (-0.26 - 0.28)	0.11 (-0.26 - 0.29)
Domain 2 The why and how of change in behavior	0.02 (-0.28 - 0.28)	-0.03 (-0.28 - 0.27)	0.02 (-0.28 - 0.28)
Domain 3 The whole consultation	-0.12 (-0.33 - 0.30)	-0.16 (-0.32 - 0.27)	-0.08 (-0.30 - 0.27)
Domain 4 Talk about targets	-0.18 (-0.37 - 0.31)	-0.23 (-0.36 - 0.28)	-0.18 (-0.36 - 0.30)
BECCI mean sum score	-0.05 (-0.26 - 0.25)	-0.09 (-0.25 - 0.23)	-0.04 (-0.25 - 0.24)
Consultation length	0.71 (-2.51 - 5.38)	0.33 (-3.34 - 4.69)	1.04 (-1.95 - 6.30)

17 cases were excluded due to missing data.

These analyses reveal no significant differences between the two types of prevention consultations (model 1), even after correcting for sociodemographics, smoking, alcohol use and physical activity (model 2, 3). None of the interaction terms added to model 3 were statistically significant (results of interaction analyses not shown). The regression coefficients are depicted in Table 3. The non-significant coefficients range was from -0.03 to -0.23. Hence, differences between the groups are small. After adjustment for sociodemographics and self-reported current lifestyle behavior the coefficients hardly change. The unexplained variance on practice nurse level (highest level) ranged from 0.044 to 0.066 for Domain 1 (model 1, 3, 2), from 0.066 to 0.80 for Domain 2, from 0.052 to 0.086 for Domain 3, from 0.081 to 0.138 for Domain 4, from 0.052 to 0.084 for BECCI mean sum score and from 30.02 to 32.62 for the consultation length. The intraclass correlation (ICC) on the four BECCI domains and BECCI mean sum score ranged from 0.10 to 0.15 for model 1.

Our results show no significant association between the type of consultation and speaking time (odds ratio = 1.3; 95% CI = 0.5 - 3.7). The unexplained variance on practice nurse level ranged from 1.336 to 1.542 (model 1 and 2) for speaking time. The ICC on speaking time is 0.29 for model 1.

## Discussion

This study is, as far as we know, the first to examine whether and how PNs apply MI techniques in real-life primary care consultations and if they adjust their MI skills to the type of prevention consultation. PNs do appear to apply MI techniques, but only to a moderate level. This is in line with previous findings suggesting that MI skills are not easily applicable in daily practice. Heinrich [28] found a limited use of MI, Voogdt-Pruis [39] concluded that within cardiovascular prevention PNs should pay more attention to MI and Efraimsson and colleagues [40] demonstrated that nurses rarely used MI techniques in their smoking cessation communication with patients. In the present study, differences in the use of MI techniques may also be due to differences in the content and extensiveness of training in MI which all PNs went through.

As mentioned before, we found that PNs use MI techniques with substantial variation between the four domains. PNs demonstrate sensitivity to talking about other issues than behavior change, encourage the patient to talk about current behavior or status quo and are sensitive to the patients’ concerns and understanding when providing information. In contrast, PNs particularly fail to meet the use of empathic statements when the patient talks about behavior change, often forget to summarize what the patient says, to ask patients about their willingness to talk about their behavior and do not always acknowledge the challenges of behavior change. Furthermore, although the majority of the PNs meet the required MI speaking time, in about one sixth of the consultations PNs talked more than half of the time.

No differences were found in the use of MI between primary and secondary prevention consultations. The gain to help patients in primary consultations by preventing complications may equal the necessity to help the disease from aggravating in secondary prevention, both requiring MI. If so, MI has relevance for primary and secondary consultations.

Our definition of primary and secondary prevention consultations is in line with previous studies [39, 41, 42]. In research on prevention, however, other definitions of primary and secondary prevention are also used. For example, Saxena, Jane-Llopis and Hosman [43] among others [44], distinguish primary, secondary and tertiary prevention in which secondary and tertiary prevention overlap with our primary and secondary prevention, respectively. To avoid ambiguity regarding the opposite or sometimes overlapping definitions of primary and secondary prevention, we plea for a more universal nomenclature.

### Strengths and limitations

Our results are based on observational data instead of self-reported data only. Another major strength is that consecutive patients were approached and recruited during a regular visit to their PN and were not selected for any type of condition. Moreover, patients were not notified in advance about the study and when asked for participation in the waiting room, only 6% refused to participate. We thus got a realistic insight into the application of behavioral change techniques in usual daily practice.

There are also some limitations. First, the length of the MI training differed substantially between PNs. Post-hoc analysis did not reveal a positive influence of the length of the training. Our observations show a limited use of MI techniques. But, some BECCI-items are only completed when it is applicable within the context. MI focuses on resolving ambivalence between desired behavior and actual behavior and on enhancing intrinsic motivation to behavioral change. When medical parameters show normal levels or patient’s lifestyle appears healthy, some MI techniques may be redundant. Furthermore, although there is evidence for the effectiveness of MI there are many other approaches to behavior change that show equally effective outcomes [24, 25]. Besides, motivation is only one of a range of factors influencing behavior [24].

### Implications for future research and clinical practice

In conformity with previous studies [28, 45, 46], our study suggests that training is not enough for acquiring MI skills. Teaching of MI techniques has been shown before to influence practitioner’s behavior [30, 45, 47], but many practitioners tend to return to old counselling habits after a few months [24, 45, 46]. Though additional training might strengthen and maintain the new counseling skills, training needs to focus on enhancing new counseling behavior consistent with MI and suppressing old counseling behavior that is inconsistent with MI [28]. Furthermore, all members of a medical practice need to be motivated to change and to have a shared understanding of the meaning of an approach [46]. Besides, it is important that health care providers are supported by their supervisor [48] and colleagues [30, 34]. We suggest that health care providers, like PNs, who want to acquire MI skills follow an extensive training with sufficient follow-up. Besides continuing education, the training could be extended by video-feedback [31] and feedback based on participating observation.

Lastly, an explanation for the insufficient MI use may be that the PNs also have to (prove to) adhere to clinical guidelines at the same time [39, 49]. It is possible that PNs find it hard to combine these guidelines with MI. Clinical practice guidelines demand PNs to meet certain task requirements, but PNs also want to take the patients’ motivation into account as part of MI. This may be contradictory. Future research could investigate this hypothesis.
